# Intra-hospital preventive principles to protect frontline healthcare workers to overcome pandemic COVID-19 in Taiwan

**DOI:** 10.1186/s13054-020-02983-7

**Published:** 2020-06-11

**Authors:** Ting-Wan Tan, Chih-Ming Chang, Man-Na Chang

**Affiliations:** 1 Department of Nursing, Hsinchu MacKay Memorial Hospital, No.690, Sec. 2, Guangfu Road, Hsinchu, 30071 Taiwan; 2 Department of Healthcare Management, Yuanpei University of Medical Technology, Hsinchu, Taiwan

## Introduction

Frontline healthcare workers have faced huge challenge and psychological stress from the pandemic COVID-19 since December 2019. Most of Taiwan’s healthcare facility has reported “Zero” infected cases in frontline healthcare workers through caring for COVID-19 confirmed cases. Implementing effective intra-hospital preventive principles to protect frontline healthcare workers’ physical and psychological well-being to overcome pandemic of COVID-19 is crucial.

## Establish appropriate control of personal protective equipment

The hospital supply department has controlled and calculated all the epidemic supplies weekly including the materials, equipment, and supplies that will be necessary to be used during the epidemic, and to determine to order extra supplies earlier than planned to maintain stock levels. Every department has implemented a rationing system to distribute surgical facial mask weekly, to be equitable for every healthcare workers. Every hospital department has filled a “stock card” when utilizing pandemic prevention and protection supplies, to prevent unnecessary waste or inappropriate usage of supplies and to alleviate acute shortage of materials, which could improve the management of supply chain to ensure healthcare workers have the resources to manage the pandemic crisis.

## Establish standardized rational use of epidemic prevention PPE to provide care and services

All healthcare workers have been organized to the standardized rational use of PPE sets, in which a healthcare professional will put on full PPE sets while performing PCR test or direct care and treatment. Hospital orderlies will wear surgical gloves and medical masks to handling samples and specimen to laboratory with appropriate leak-proof container to prevent contaminations. CSSD operators will put on disposable face shield, medical mask, headwear, waterproof gloves, and waterproof gown to cleanse medical equipment and surgical instruments, to prevent overuse and shortage of PPE sets (Table 1) [1].

**Table 1:** Standardized rationale use of epidemic prevention PPE [1]

Healthcare Settings	Target personnel	Activity	Respirators		Gloves		Gowns			Eye Protection		Headwear
			Medical Mask	Respiratory N95	Surgical Gloves	Water Proof Gloves	Fluid Repellent Gown	Fluid Resistant Gown	Water Proof Apron	Goggles	Face Shields	
Administrative Areas	All staffs	Administrative Tasks	V									
Screening Area	Staffs	Screening (Temperature/TOCC)	V									
Outpatient Clinics (Consultation room)	Healthcare Workers	Consultation Physical Examinations	V									
Triage	Healthcare Workers	Preliminary Screening	V									
	Orderlies	Transport Patients	V		V							
Other Area of patient transit (Wards)	Healthcare Workers	Provide direct care		V	V		V			V		V
	Orderlies	Transport Patients		V	V		V					V
Laboratory	Orderlies	Handling samples and specimen	V		V							
Temporary Isolation Area (Wards)	Healthcare Workers	Provide direct care		V	V			V			V	V
	Cleaners	Cleaning Environment		V	V			V			V	V
Central Sterile Services Department (CSSD)	CSSD Operators	Cleaning Medical Equipment/Supplies	V			V			V		V	V

## Ongoing and update educations for all frontline healthcare workers

During early epidemic of COVID-19, every nursing unit has provide adequate and intense education and training online learning resources regarding to the adequate steps of putting on PPEs, hand hygiene steps, route of transfer for suspected or confirmed COVID-19 cases, appropriate procedure of handling specimen with suitable container, and intense environment cleaning procedures of which has all record as learning videos and posted on hospital learning website and LINE group for all healthcare providers (including orderlies and cleaners). All the frontline healthcare providers have been arranged to learning from online courses at least 80% of completion rates as regulated by infectious specialist in hospital, which helps healthcare providers to get better understanding of COVID-19.

## Strictly standardized routine environmental cleaning of every nursing unit in the hospital

Every nursing unit has arranged cleaning schedule twice daily, to use 500 ppm NaHCO3 to cleanse frequently touched surfaces such as door handles, computer screen, keyboards, computer mouse, desks, chairs, and switches in nursing station and break room, to disinfect the workplace to prevent the spread of COVID-19.

Hospital cleaning staffs have to disinfect all areas which have contacted to suspected or confirmed COVID-19 cases, have worn full PPE while cleaning, and used appropriate detergent solution 5000 ppm NaHCO3 with disposable clothes to disinfect all hard and soft surfaces. Fluorescent marker method and ATP bioluminescence were being used to detect environment cleanliness.

## Assemble all mobile medical equipment to CSSD department

The hospital has to assemble mobile medical equipment to CSSD after use from patients, to ensure assemble in a flawless environment and follow cleaning and disinfectant protocols to cleanse the equipment; this would ultimately be an effective cleaning system with all quality practice to prevent from contaminations.

## Digital technology to observe isolation patients

The hospital has an innovative use of camera technology to observe patients in isolation room for 24 h; physicians and nurses were able to monitor and converse with patients via camera technology individually. Healthcare workers only perform face to face nursing care and treatment when necessary to prevent unnecessary usual contact to prevent contaminations [2].

## Banned to rotate across the unit/wards

Frontline healthcare workers (including orderlies, cleaning, CSSD staffs) were separated into two groups to work in fixed unit especially isolation wards. Nurses, orderlies, and cleaning staffs are banned to rotated working in different units to reduce hospital-acquired transmission. If healthcare workers have to rotate across the unit, they will rotate at least in a monthly schedule to prevent the spread of viruses.

Every healthcare worker requires monitoring of temperate and URI symptoms every 8 h. While reported temperature higher than 37.5 °C or presence of URI symptoms will be reported to the hospital’s clinical infectious and occupational health teams, staffs will be necessarily followed up in a COVID-19 special clinic to follow-up with the PCR test and will be home quarantined till the PCR test turns out to be negative.

## Provide emotional support for healthcare workers

**Fig. 1:**
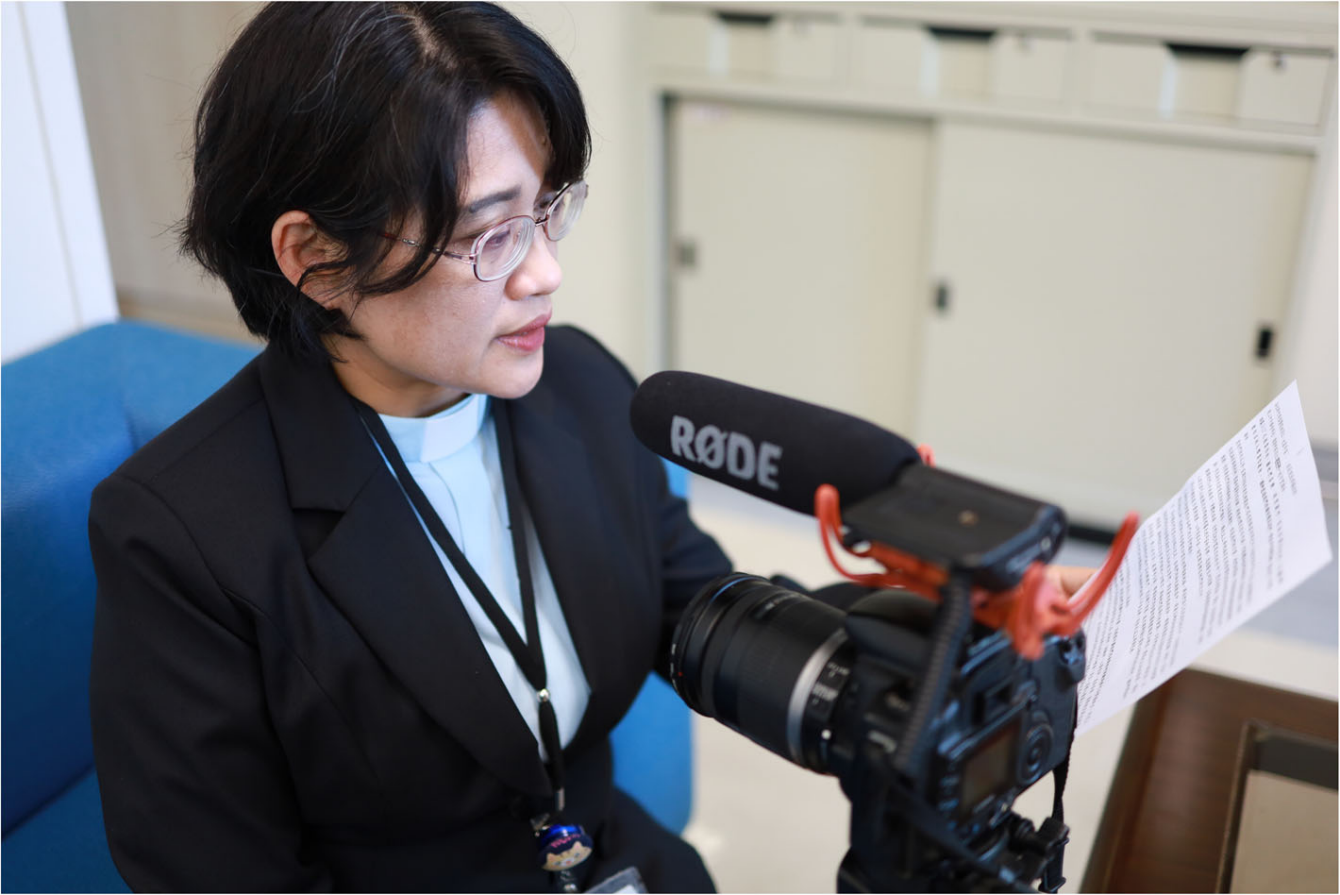
Hospital priest has prayed together with all healthcare workers weekly for blessing

**Fig. 2:**
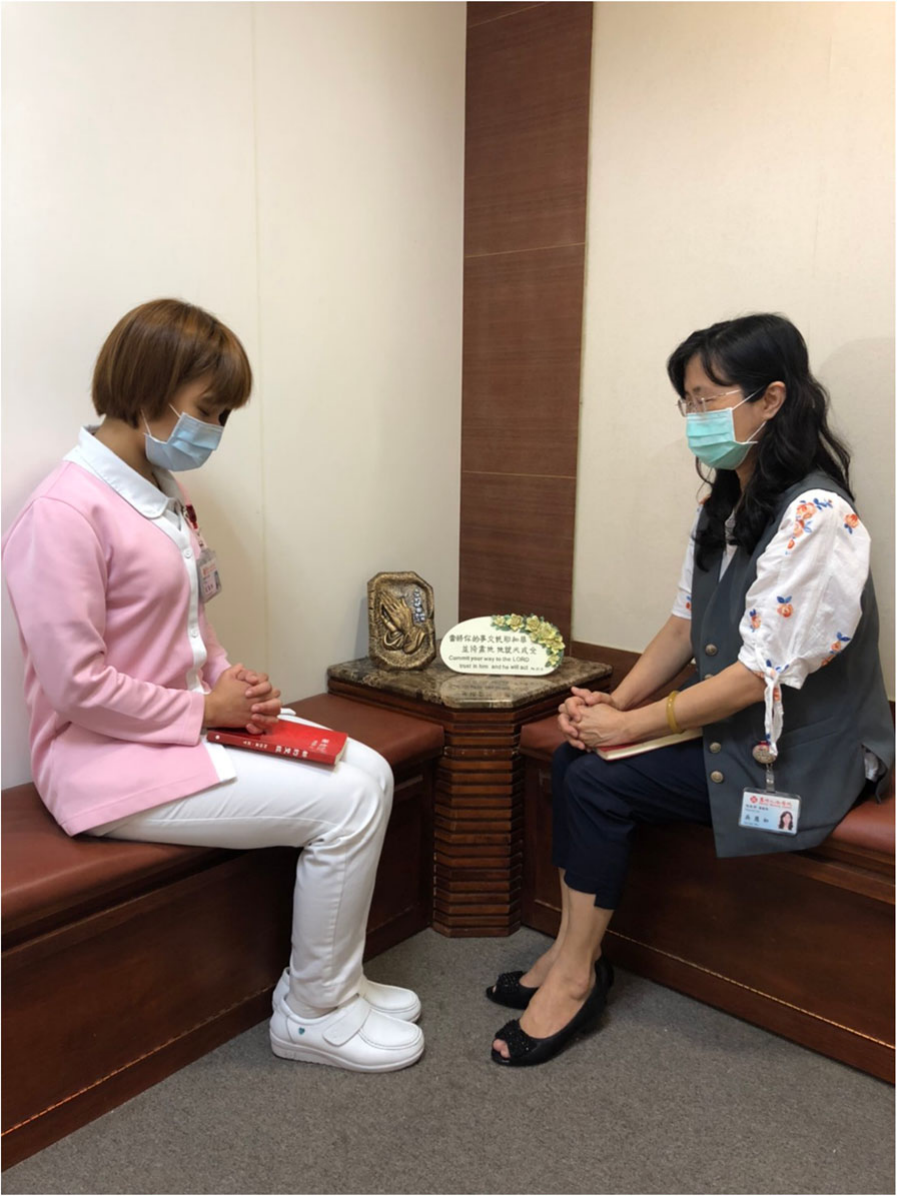
Hospital spiritual care workers providing psycho-social-spiritual services for frontline healthcare workers

Hospital priest has prayed together with all healthcare workers weekly through hospital broadcasting services, which could relieve emotional strain and physical exhaustion for frontline healthcare workers (Fig. 1) [3]. Hospital spiritual care workers have provided physiological supports through telehealth services, including LINE apps, online resources, and virtual support to sustain and monitor healthcare workers’ mental health status and to provide empathy and support (Fig. 2). This aims to optimize healthcare workers’ psychological needs during the COVID-19 pandemic attack.

## Conclusion

COVID-19 has rapidly spread across the world since December 2019. The outbreak has caused huge psychological distress for frontline healthcare workers; additional ongoing and clear information and appropriate knowledge through formal or informal training are crucial for self-care. Institutional supportive care and seeking peer support are extremely important to express stress and anxiety feelings and to maintain emotional well-being for healthcare workers to provide care for COVID-19 patients.

## Data Availability

Not applicable.
